# Longitudinal changes in BMI and obesity across COVID-19 pandemic periods among underrepresented populations: An analysis of the All of Us dataset

**DOI:** 10.1371/journal.pone.0360745

**Published:** 2026-10-27

**Authors:** Abdul-Hanan Saani Inusah, Huiyi Xia, Atena Pasha, Zhenlong Li, Andrew T. Kaczynski, Xiaoming Li, Shan Qiao

**Affiliations:** 1 Department of Health Promotion, Education and Behavior, Arnold School of Public Health, University of South Carolina, Columbia, South Carolina, United States of America; 2 South Carolina SmartState Center for Healthcare Quality, Arnold School of Public Health, University of South Carolina, Columbia, South Carolina, United States of America; 3 Geoinformation and Big Data Research Lab, Department of Geography, The Pennsylvania State University, University Park, Pennsylvania, United States of America; Mayo Clinic College of Medicine and Science, UNITED STATES OF AMERICA

## Abstract

**Introduction:**

The COVID-19 pandemic significantly affected physical activity and lifestyle, potentially increasing Body Mass Index (BMI) and obesity risk. We examined changes in BMI and obesity across Pre-Pandemic, Pandemic Peak, and Peri-Pandemic periods and identified sociodemographic correlates.

**Methods:**

We analyzed longitudinal electronic health record data from the All of Us Research Program for adults ≥18 years with at least one BMI record in each period (N = 38,632). Periods were defined as Pre-Pandemic (Jan 1, 2018–Mar 12, 2020), Pandemic Peak (Mar 13, 2020–Dec 31, 2021), and Peri-Pandemic (Jan 1, 2022–Oct 31, 2023). BMI was modeled using linear mixed-effects models and obesity (BMI ≥ 30) using GEE logit models, adjusting for age, sex, race, ethnicity, income, and employment. A sensitivity analysis was conducted by expanding eligibility to participants with BMI data in at least two periods (N = 83,698).

**Results:**

Mean BMI increased from 30.05 (SD = 6.67) Pre-Pandemic to 30.14 (SD = 6.76) during the Pandemic Peak, then declined to 29.96 (SD = 6.66) Peri-Pandemic; obesity prevalence followed a similar pattern (43.1% → 43.4% → 42.8%). In adjusted models, the linear time effects were positive and the quadratic terms negative for both outcomes (BMI: β = 0.218 and −0.018; obesity: aORs = 1.053 and 0.996; all p < 0.001). Female, Black/African American, and Hispanic/Latino participants had higher adjusted BMI and greater odds of obesity than their respective reference groups, while higher income was associated with lower BMI and obesity odds. Significant time × subgroup interactions were observed, with age and employment status showing the most consistent effects across both outcomes.

**Conclusion:**

BMI and obesity displayed statistically detectable but modest changes across the pandemic-defined periods. Persistent adjusted differences by sex, race, ethnicity, and income highlight the relevance of equitable access to obesity-prevention resources.

## Introduction

The COVID-19 pandemic disrupted daily routines worldwide, leading to significant changes in health-related behaviors. Lockdowns, remote schooling/working, and travel restrictions limited access to physical activity opportunities and changed dietary patterns across all age groups [1,2]. These behavioral disruptions, along with increased stress and social isolation, created an environment that contributed to weight gain and worsened the existing obesity epidemic [1,3,4]. For underserved populations, this closure also reduced resources that often provide access to meals and emotional support [5,6]. These public health measures, though necessary for curbing viral spread, had unintended consequences on population-level BMI trends and metabolic health, including elevated rates of obesity [7,8].

Obesity was a global public health concern even before the pandemic. In the U.S., more than 42% of adults were classified as obese in 2017–2018, and these rates would continue to rise in the following years [9,10]. The COVID-19 pandemic further exacerbated these trends as studies have shown significant increases in average BMI and obesity rates during the pandemic, with both adults and children experiencing faster weight gain compared to pre-pandemic periods [8,11,12]. In this regard, longitudinal analyses revealed average monthly weight gains of 1–1.5 pounds during shelter-in-place mandates [13,14].

However, these changes have not been distributed evenly, as racial and ethnic minority groups and individuals with lower socioeconomic status experienced a disproportionate burden of both COVID-19 outcomes and obesity-related risk factors [15,16]. The disparities reflect long-standing structural inequities in access to healthcare, healthy foods, safe neighborhoods, and economic stability. For instance, Black and Hispanic populations, already more likely to have obesity, also faced greater job insecurity, income loss, and limited access to recreational spaces during the pandemic [17,18]. Simultaneously, these groups experienced higher rates of COVID-19 morbidity and mortality, further compounding their health risks [19,20]. These socioeconomic stressors, psychological distress, and food insecurity are all established correlates of increased BMI [21–23].

Despite growing literature on pandemic-related weight changes, existing studies often suffer from key limitations that restrict their policy relevance and scientific generalizability of historically marginalized groups. Many have focused narrowly on comparing only two time points (e.g., pre- versus during-pandemic), without examining how weight trends evolved after public health restrictions were lifted [8,24]. Others have relied on convenience samples, geographically limited datasets, or clinical cohorts, making it difficult to draw conclusions about national trends or diverse populations [11,12]. Moreover, prior studies often lacked detailed sociodemographic factors (e.g., sex, employment status, income, race/ethnicity) that are critical to understanding disparities in obesity outcomes. This gap is critical, given that the pandemic may have differentially affected health behaviors and obesity risk among historically marginalized groups.

To address these limitations, this study aims to provide a comprehensive longitudinal analysis of BMI and obesity trends across Pre-Pandemic, Pandemic Peak and Peri-Pandemic periods using the National Institutes of Health All of Us Program, and to identify sociodemographic factors, such as age, sex, race, ethnicity, income, and employment status, associated with differences in BMI and obesity over time. Focusing on vulnerable and historically underrepresented groups, this analysis offers important insight into the health implications of the COVID-19 pandemic.

## Methods

### Data source and participants

We conducted a retrospective cohort study using data from the All of Us (AoU) Program (Curated Data Repository Version 8, Controlled Tier). The AoU program is a nationwide precision medicine initiative that aims to collect diverse health data from at least one million participants across the United States [25]. This dataset includes electronic health record (EHR) and survey information for a broad, geographically and demographically diverse cohort. Approximately 78% of participants belong to groups historically underrepresented in biomedical research, including racial and ethnic minorities, individuals with lower income and educational attainment, and sexual and gender minorities [26]. EHR data in AoU are collected by participating health care provider organizations and submitted to the program’s Data and Research Center, which harmonizes and curates the data for research use [25]**.** Prior validation studies have confirmed the reliability of these data, including replication of known clinical patterns and treatment pathways, thereby supporting the dataset’s quality and utility for population health research [26].

Data were accessed from the All of Us Research Program on December 6, 2025. All data were de-identified, and the authors did not have access to identifiable participant information. All adults aged 18 years or older with available BMI measurements in each of the three pandemic-related time periods were eligible for inclusion. These periods were defined as: Pre-Pandemic (January 1, 2018-March 12, 2020), Pandemic Peak (March 13, 2020-December 31, 2021), and Peri-Pandemic (January 1, 2022-October 31, 2023). Although the worldwide COVID-19 pandemic did not end officially until May 2023 [27], we defined the Peri-Pandemic period beginning January 1, 2022 to reflect major behavioral and contextual shifts relevant to obesity research, including widespread vaccine availability, relaxation of restrictions, reopening of schools and workplaces, and resumption of in-person activities. This is consistent with observations from Ioannidis (2022) and the CDC Museum COVID-19 Timeline indicating that population behaviors and mobility patterns in late 2021 began returning toward pre-pandemic norms [28,29].

Given that BMI and obesity values were derived from EHR and could be affected by changes in healthcare utilization, the primary analytic sample was restricted to participants with at least one BMI measurement in each of the three periods. This criterion standardized data availability across periods but may preferentially retain participants with continued healthcare engagement and does not eliminate selection bias. To reduce the influence of extreme age outliers, participants with age outside the 2^nd^ to 97^th^ percentiles were excluded (n = 97). The final primary analytic sample included 38,632 participants.

### Outcome measures

Two outcome measures were examined: BMI as a continuous variable and obesity as a binary variable. BMI was defined as weight in kilograms divided by height in meters squared, and BMI values were obtained exclusively from EHR-derived height and weight measurements. Self-reported survey BMI was not included. For participants with multiple BMI measurements within a single calendar year, all BMI values for that year were averaged to produce one representative annual BMI value per person. This annual average was used to reduce intra-individual measurement variability and provide a stable estimate of BMI for each calendar year. Using these annual BMI values, we defined obesity status as a binary indicator (obese vs. non-obese). Obesity was classified as an average BMI ≥ 30 kg/m² for the year, consistent with standard clinical definitions [30]. We used BMI rather than obesity diagnosis codes because diagnosis coding captures only clinically documented obesity and would not allow us to evaluate population-level weight trajectories across time. Each participant therefore had a BMI value and corresponding obesity classification for each of the three time periods. For 2020, BMI values prior to March 13 were assigned to the Pre-Pandemic period and those on or after March 13 were assigned to the Pandemic Peak period, ensuring that annual averages did not combine measurements across phases.

### Covariates

Sociodemographic covariates included age, sex, race, ethnicity, household income, and employment status. Participants who did not respond to survey-based demographic questions or selected “Prefer not to answer” or “Skip” were categorized as “Unknown” for the corresponding variable.

Age was categorized as <50 years and  ≥ 50 years for analyses. Age 50 was used as the cutoff because it reflects midlife in epidemiologic and cardiometabolic research and provided a relatively balanced distribution in the study sample.

In the AoU dataset, race, ethnicity, and annual household income are derived from EHR or participant self-report, depending on the contributing healthcare organization. Several race categories contained very small cell sizes, including American Indian/Alaska Native, Middle Eastern/North African, and Native Hawaiian/Other Pacific Islander. For descriptive purposes, we present all available race categories in Table 1, including participants whose race was reported as unknown. For regression analyses, we combined smaller race groups and individuals with unknown race into a single Asian/Other/Unknown category to increase analytic stability and maintain adequate power.

**Table 1:** Sociodemographic characteristics and descriptive trends in BMI and obesity across Pre-Pandemic, Pandemic Peak and Peri-Pandemic periods (N = 38,632).

Variables	Overall, n (%)	BMI, mean (SD)				Obesity prevalence, n (%)			
		Pre-Pandemic	Pandemic Peak	Peri-Pandemic	p-value (ANOVA)	Pre-Pandemic	Pandemic Peak	Peri-Pandemic	p-value (GEE)
Total sample	38,632 (100.0)	30.05 (6.67)	30.14(6.76)	29.96(6.66)		16,666 (43.1)	16,765 (43.4)	16,524 (42.8)	
Overall age, mean (SD), years	50.6 (13.7)								
Age, median [range], years	52.0 [21.0, 76.0]								
**Age group**									
Younger adults (<50 years)	17,354 (44.9)	30.61 (7.15)	30.86 (7.24)	30.86 (7.15)	< 0.001	8,115 (46.8)	8,366 (48.2)	8,411 (48.5)	< 0.001
Older adults (≥50 years)	21,278 (55.1)	29.52 (6.14)	29.37 (6.19)	29.15 (6.11)		8,551 (40.2)	8,399 (39.5)	8,113 (38.1)	
**Sex**									
Male	12,844 (33.2)	29.38 (5.58)	29.30 (5.65)	29.19 (5.61)	< 0.001	4,913 (38.3)	4,862 (37.9)	4,745 (36.9)	< 0.001
Female	25,788 (66.8)	30.32 (7.09)	30.41 (7.17)	30.29 (7.09)		11,753 (45.6)	11,903 (46.2)	11,779 (45.7)	
**Race**									
White	23,044 (59.7)	29.24 (6.39)	29.24 (6.46)	29.18 (6.39)	< 0.001	8,762 (38.0)	8,747 (38.0)	8,717 (37.8)	
Black/African American	6,641 (17.2)	32.67 (7.27)	32.74 (7.34)	32.47 (7.32)		3,950 (59.5)	3,997 (60.2)	3,893 (58.6)	0.061
Asian	873 (2.3)	25.91 (4.69)	25.88 (4.77)	25.91 (4.74)		155 (17.8)	142 (16.3)	149 (17.1)	0.584
American Indian/Alaska Native	255 (0.7)	31.37 (7.01)	31.60 (7.20)	31.53 (6.87)		138 (54.1)	146 (57.3)	142 (55.7)	0.157
Middle Eastern/North African	165 (0.4)	29.01 (6.15)	28.90 (5.91)	28.96 (5.99)		58 (35.2)	54 (32.7)	56 (33.9)	0.858
Native Hawaiian/Other Pacific Islander	29 (0.1)	33.57 (5.51)	33.45 (5.81)	33.79 (6.77)		19 (65.5)	20 (69.0)	18 (62.1)	0.532
Other	7,039 (18.2)	30.53 (6.19)	30.66 (6.28)	30.47 (6.24)		3,369 (47.9)	3,431 (48.7)	3,336 (47.4)	0.544
Unknown	586 (1.5)	29.39 (6.53)	29.34 (6.64)	29.06 (6.52)		215 (36.7)	228 (38.9)	213 (36.3)	0.756
**Race (regression categories)**									
White	23,044 (59.7)	29.24 (6.39)	29.24 (6.46)	29.18 (6.39)	< 0.001	8,762 (38.0)	8,747 (38.0)	8,717 (37.8)	
Black/African American	6,641 (17.2)	32.67 (7.27)	32.74 (7.34)	32.47 (7.32)		3,950 (59.5)	3,997 (60.2)	3,893 (58.6)	0.060
Asian/Other/ Unknown	8,947 (23.2)	30.01 (6.26)	30.11 (6.36)	29.94 (6.30)		3,954 (44.2)	4,021 (44.9)	3,914 (43.7)	0.640
**Ethnicity**									
Non-Hispanic/Latino	31,524 (81.6)	29.95 (6.76)	29.98 (6.84)	29.87 (6.77)	< 0.001	13,415 (42.6)	13,431 (42.6)	13,302 (42.2)	
Hispanic/Latino	6,153 (15.9)	30.33 (5.99)	30.43 (6.03)	30.27 (6.01)		2,876 (46.7)	2,948 (47.9)	2,861 (46.5)	0.940
Other/Unknown	955 (2.5)	29.66 (6.64)	29.65 (6.75)	29.38 (6.64)		375 (39.2)	386 (40.4)	361 (37.8)	0.230
**Annual Income Level**									
<$25,000	7,487 (19.4)	31.83 (7.22)	31.98 (7.31)	31.69 (7.28)	< 0.001	4,078 (54.5)	4,141 (55.3)	4,035 (53.9)	
$25,000-$50,000	4,867 (12.6)	31.39 (6.97)	31.46 (6.98)	31.32 (6.96)		2,576 (52.9)	2,591 (53.2)	2,546 (52.3)	0.583
$50,000-$100,000	8,000 (20.7)	30.03 (6.52)	30.01 (6.60)	29.96 (6.56)		3,470 (43.3)	3,453 (43.2)	3,444 (43.0)	0.377
>$100,000	11,200 (29.0)	28.08 (5.70)	28.06 (5.80)	28.08 (5.70)		3,393 (30.3)	3,383 (30.2)	3,411 (30.5)	0.043
Unknown	7,078 (18.3)	30.14 (6.46)	30.18 (6.53)	29.96 (6.49)		3,149 (44.5)	3,197 (45.2)	3,088 (43.6)	0.638
**Employment Status**									
Employed	17,619 (45.6)	29.82 (6.61)	29.95 (6.71)	29.97 (6.64)	<0.001	7,370 (41.8)	7,492 (42.5)	7,539 (42.8)	
Unemployed	21,013 (54.4)	30.16 (6.66)	30.12 (6.73)	29.88 (6.66)		9,296 (44.2)	9,273 (44.1)	8,985 (42.8)	<0.001

Mean (SD) for BMI and n (%) for obesity are reported. BMI p-values are based on repeated-measures ANOVA. Obesity p-values are based on bivariate GEE models evaluating subgroup differences across the three study periods.

For regression analyses, ethnicity was categorized as Non-Hispanic/Latino, Hispanic/Latino, or Other/Unknown. The Other/Unknown category comprised participants whose ethnicity information was not classified as either Non-Hispanic/Latino or Hispanic/Latino in the analytic dataset, including other, unknown, or otherwise unclassified responses.

Annual household income was grouped into four categories (<$25,000, $25,000–$50,000, $50,000–$100,000, and >$100,000), and the ‘Unknown’ category was retained as a separate group because its frequency was sufficiently large. Employment status was categorized as employed versus unemployed. These variables were included as covariates in the regression models to control for potential confounding.

### Statistical analysis

We conducted descriptive analyses to examine the trends of BMI and obesity in the whole sample and by demographic subgroups. The overall differences between subgroups by each demographic variable were tested using repeated-measures ANOVA (for BMI) and bivariate generalized estimating equation (GEE) models (for obesity). We then employed multivariable regression models to assess changes in BMI and obesity across the three time periods. A linear mixed-effects model was used for BMI (continuous outcome), and a GEE with a logit link was used for obesity (binary outcome). Time was modeled as both categorical (Pre-Pandemic, Pandemic Peak and Peri-Pandemic) and continuous with a quadratic term to capture potential non-linear trends.

The multivariable models were adjusted for age, sex, race, ethnicity, household income level, and employment status. Two-way interaction terms between time and each sociodemographic factor were included to test for subgroup differences over time. The mixed-effects model incorporated a person-specific random intercept to account for repeated measures within individuals, while the GEE used an exchangeable working correlation structure to account for within-person correlation.

Results from the mixed-effects model were reported as β coefficients (adjusted mean differences) with 95% confidence intervals (CIs), and GEE results were reported as adjusted odds ratios (aORs) with 95% CIs. Two-sided p-values < 0.05 were considered statistically significant; values below 0.001 were reported as p < 0.001. All analyses were conducted using R software (version 4.2.0).

### Sensitivity analysis

To assess robustness to the requirement for BMI data in all three periods, we repeated the analysis using a less restrictive criterion of BMI data in at least two of the three periods. This expanded the cohort from 38,632 to 83,698 participants. The same model types and covariates were applied for directly comparable variables. Directly comparable results are reported in S1 Table.

### Ethics

This study was conducted using de-identified data from the AoU Research Program, which operates under a central institutional review board (IRB) approval. All participants in AoU provided informed consent at enrollment. As this study used de-identified secondary data, no additional IRB approval was required.

## Results

### Sample characteristics

This analysis includes 38,632 adults aged 18 years and older with at least one BMI record in each of the three designated time periods. As shown in Table 1, the mean baseline age was 50.6 years (SD = 13.7). The majority of participants were female (66.8%), White (59.7%), and non-Hispanic/Latino (81.6%). Nearly one-third of the sample reported household incomes over $100,000. Overall, 45.6% were employed and 54.4% were unemployed.

### BMI and obesity prevalence trends

As shown in Table 1, mean BMI increased from 30.05 (SD = 6.67) in the Pre-Pandemic period to 30.14 (SD = 6.76) during the Pandemic Peak, then declined to 29.96 (SD = 6.66) in the Peri-Pandemic period. Obesity prevalence followed a similar pattern, increasing from 43.1% Pre-Pandemic to 43.4% during the Pandemic Peak, and then decreasing to 42.8% in the Peri-Pandemic period. Thus, both BMI and obesity prevalence increased during the Pandemic Peak and subsequently declined to levels close to Pre-Pandemic values. Although statistically detectable in this large sample, the absolute overall changes were modest. Subgroup trajectories across age, sex, race, ethnicity, and employment are shown in Fig 1 (BMI) and Fig 2 (obesity), which visually depict these descriptive patterns over the three time periods.

**Fig 1:**
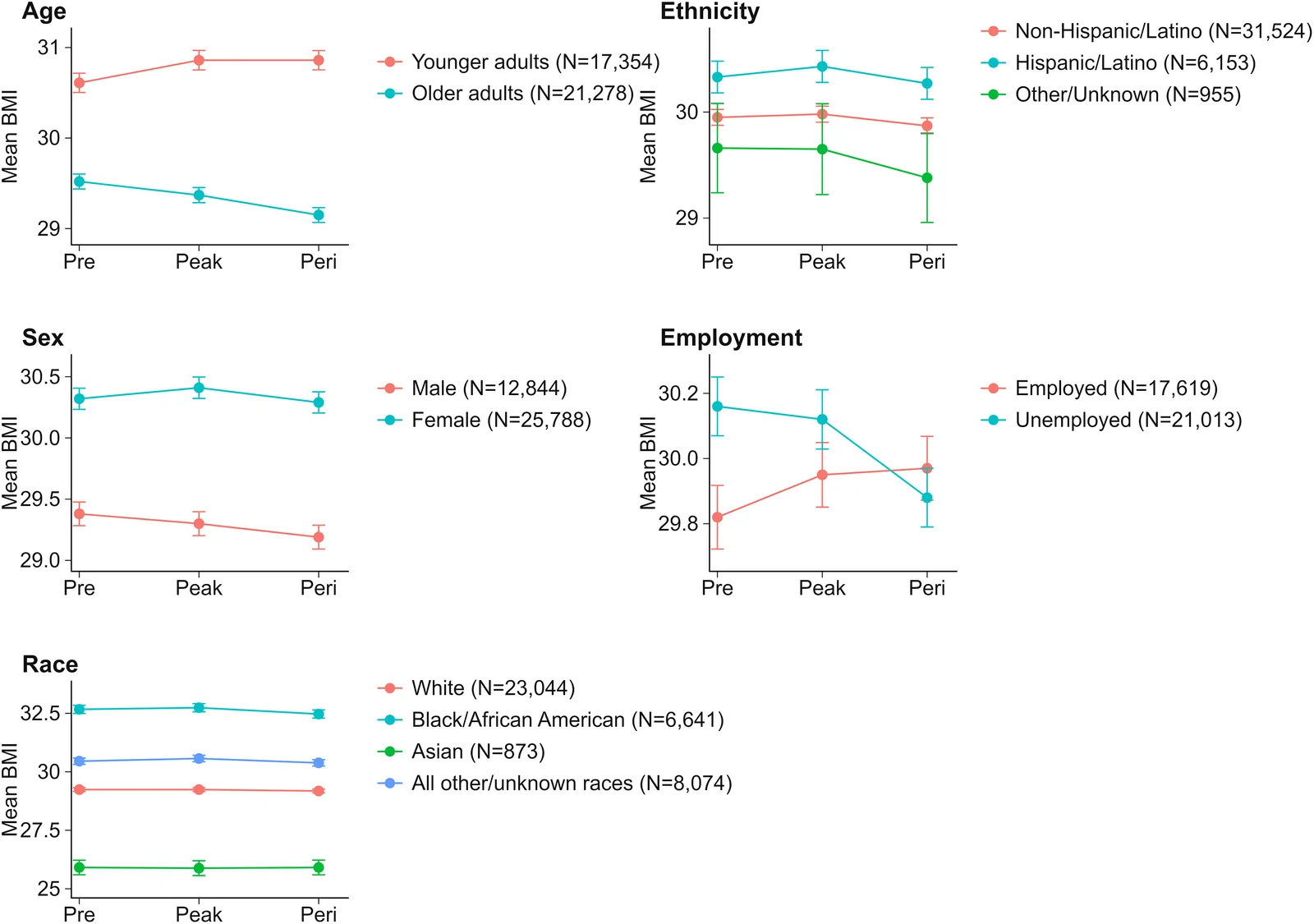
Mean BMI across sociodemographic subgroups during the Pre-Pandemic, Pandemic Peak, and Peri-Pandemic periods. Points represent mean BMI, error bars represent 95% confidence intervals, and Ns shown in the legends represent subgroup participant counts. The All other/unknown races category combines American Indian/Alaska Native, Middle Eastern/North African, Native Hawaiian/Other Pacific Islander, Other, and Unknown.

**Fig 2:**
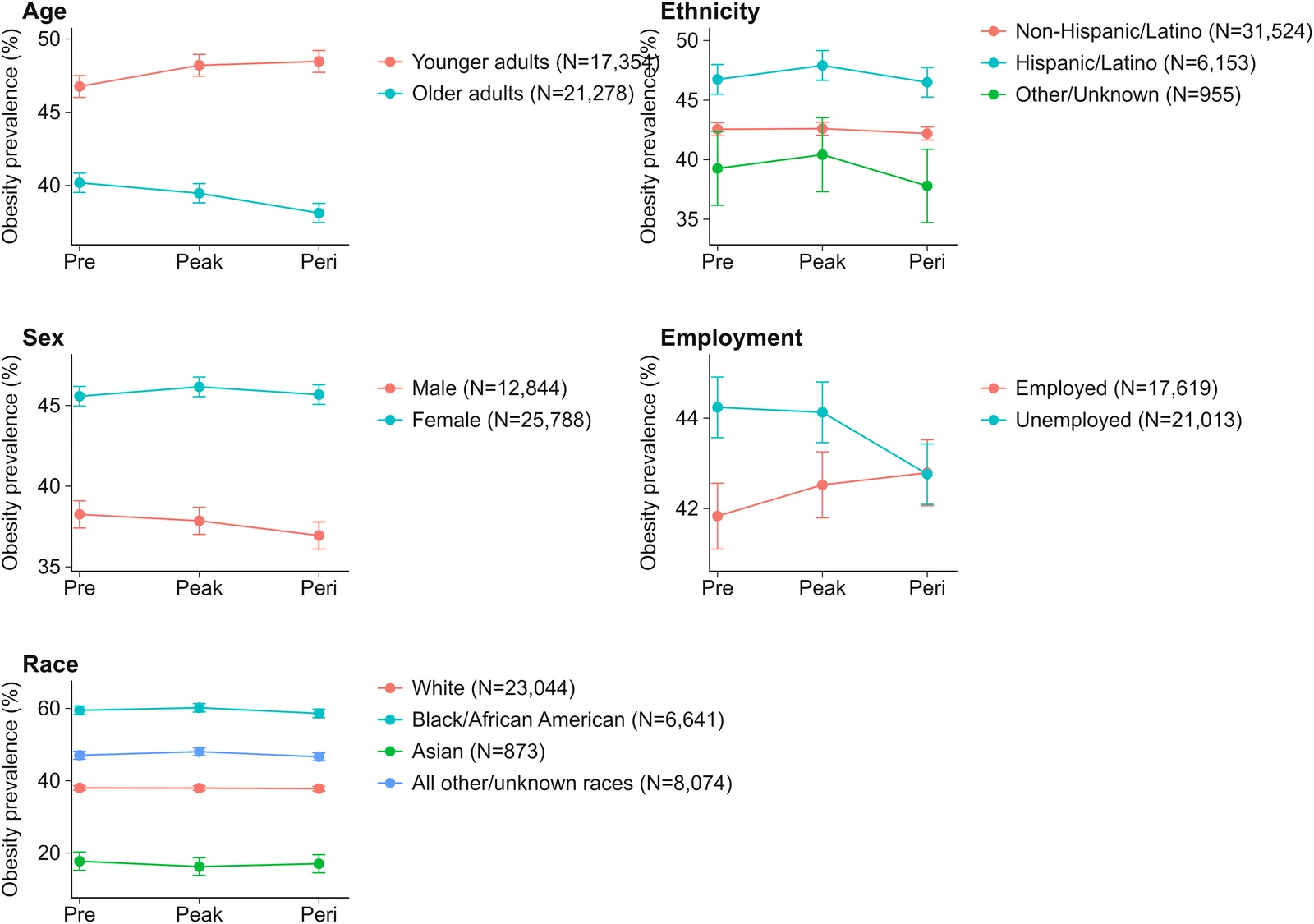
Obesity prevalence across sociodemographic subgroups during the Pre-Pandemic, Pandemic Peak, and Peri-Pandemic periods. Points represent obesity prevalence, error bars represent 95% confidence intervals, and Ns shown in the legends represent subgroup participant counts.

Age- and sex-specific descriptive differences were also observed for obesity. Among younger adults (< 50 years), obesity prevalence increased from 46.8% during the Pre-Pandemic period to 48.2% during the Pandemic Peak, and rose slightly further to 48.5% in the Peri-Pandemic period. In contrast, older adults (≥ 50 years) experienced steady declines in obesity across all three periods (40.2% → 39.5% → 38.1%). Sex patterns showed a similar divergence: men exhibited a consistent decline in obesity prevalence (38.3% → 37.9% → 36.9%), whereas women experienced an increase during the Pandemic Peak (45.6% → 46.2%) followed by a decrease in the Peri-Pandemic period (45.7%).

BMI differed significantly across the sociodemographic characteristics examined (all p < 0.001; Table 1), with higher mean BMI generally observed among younger adults, females, Black/African American participants, and participants with lower household income. Similar sociodemographic patterns were observed for obesity prevalence, which was generally higher among younger adults, females, and Black/African American participants and decreased across increasing household-income categories.

### Multivariable analysis (BMI)

#### Main effects of demographic factors.

These coefficients represent adjusted mean differences in BMI across the three periods, accounting for repeated measurements within individuals and the modeled time trend. As shown in Table 2, older adults had lower BMI than younger adults (β = −0.540, 95% CI: −0.684 to −0.396). Female participants had higher BMI than male participants (β = 0.523, 95% CI: 0.383 to 0.662). Black/African American participants had higher BMI than White participants (β = 2.461, 95% CI: 2.265 to 2.656), whereas the estimate for Asian/Other/Unknown participants was not statistically significant (β = –0.220, 95% CI: −0.471 to 0.030). Relative to Non-Hispanic/Latino participants, Hispanic/Latino participants had higher BMI (β = 0.531, 95% CI: 0.258 to 0.803).

**Table 2:** Adjusted mixed-effects regression results for BMI across the Pre-Pandemic, Pandemic Peak, and Peri-Pandemic periods (N = 38,632).

Variable	β coefficient	95%: CI	p-value
**Age**			
Younger adults (<50 years)	Reference		
Older adults (≥50 years)	−0.540	(−0.684, −0.396)	<0.001
**Sex**			
Male	Reference		
Female	0.523	(0.383, 0.662)	<0.001
**Race**			
White	Reference		
Black/African American	2.461	(2.265, 2.656)	<0.001
Asian/Other/Unknown	−0.220	(−0.471, 0.030)	0.085
**Ethnicity**			
Non-Hispanic/Latino	Reference		
Hispanic/Latino	0.531	(0.258, 0.803)	<0.001
Other/Unknown	0.307	(−0.166, 0.781)	0.204
**Annual household income**			
<$25,000	Reference		
$25,000-$50,000	−0.035	(−0.277, 0.206)	0.775
$50,000-$100,000	−1.064	(−1.287, −0.841)	<0.001
>$100,000	−2.907	(−3.125, −2.688)	<0.001
Unknown	−1.389	(−1.602, −1.176)	<0.001
**Employment status**			
Employed	Reference		
Unemployed	0.037	(−0.112, 0.185)	0.628
Time	0.218	(0.192, 0.243)	<0.001
Time squared	−0.018	(−0.021, −0.015)	<0.001
**Time interactions**			
Older adults × Time	−0.139	(−0.148, −0.130)	<0.001
Female × Time	0.020	(0.011, 0.029)	<0.001
Black/African American × Time	−0.043	(−0.056, −0.031)	<0.001
Asian/Other/Unknown race × Time	0.003	(−0.013, 0.020)	0.691
Hispanic/Latino × Time	−0.023	(−0.041, −0.005)	0.011
Other/Unknown ethnicity × Time	−0.052	(−0.083, −0.022)	0.001
Unemployed × Time	−0.057	(−0.067, −0.048)	<0.001
$25,000-$50,000 × Time	0.001	(−0.014, 0.017)	0.862
$50,000-$100,000 × Time	0.005	(−0.009, 0.020)	0.471
>$100,000 × Time	0.003	(−0.011, 0.018)	0.639
Unknown income × Time	−0.012	(−0.025, 0.002)	0.102

Higher household income was associated with lower BMI. Compared with participants earning < $25,000 annually, those earning $50,000–$100,000 had lower BMI (β = −1.064, 95% CI: −1.287 to −0.841), with a larger difference observed among those earning> $100,000 (β = −2.907, 95% CI: −3.125 to −2.688). The $25,000–$50,000 group did not differ significantly from the < $25,000 group (β = −0.035, 95% CI: −0.277 to 0.206). Employment status was not significantly associated with BMI (β = 0.037, 95% CI: −0.112 to 0.185). Fig 3 summarizes the adjusted associations for BMI and obesity.

**Fig 3:**
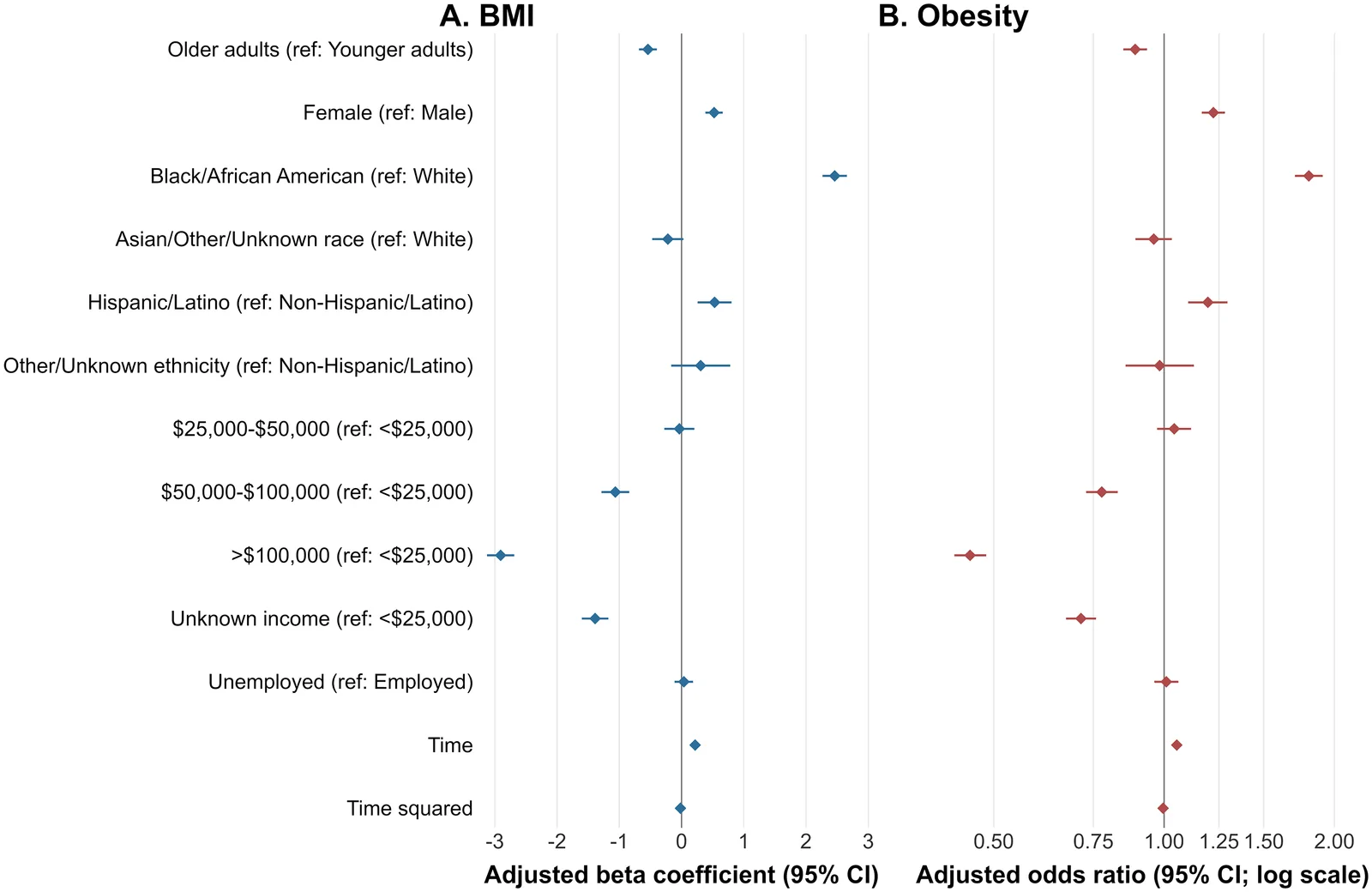
Forest plots of adjusted associations with BMI and obesity across the three study periods (N = 38,632). Panel A presents adjusted β coefficients for BMI, and Panel B presents adjusted odds ratios (aORs) for obesity. Points represent adjusted estimates and horizontal lines represent 95% confidence intervals; reference categories are indicated in the labels.

#### Time effects.

The linear time term was positive (β = 0.218, 95% CI: 0.192 to 0.243), whereas the quadratic term was negative (β = −0.018, 95% CI: −0.021 to −0.015), indicating a nonlinear temporal pattern across the three periods (Table 2).

#### Interaction effects.

As shown in Table 2, BMI changes over time differed by age, sex, Black/African American race, ethnicity, and employment. Older adults (β = −0.139, 95% CI: −0.148 to −0.130), Black/African American participants (β = −0.043, 95% CI: −0.056 to −0.031), and unemployed participants (β = −0.057, 95% CI: −0.067 to −0.048) had smaller changes over time than their reference groups. Female participants had a small positive interaction (β = 0.020, 95% CI: 0.011 to 0.029). Significant ethnicity × time interactions were observed for Hispanic/Latino participants (β = −0.023, 95% CI: −0.041 to −0.005) and participants in the Other/Unknown ethnicity category (β = −0.052, 95% CI: −0.083 to −0.022). Income interactions were not statistically significant.

### Multivariable analysis (Obesity)

#### Main effects of demographic factors.

The adjusted odds ratios represent the associations with obesity across the study periods while accounting for the modeled time trend. As shown in Table 3, older adults had lower odds of obesity than younger adults (aOR = 0.889, 95% CI: 0.847 to 0.933), whereas female participants had higher odds than male participants (aOR = 1.222, 95% CI: 1.166 to 1.281). Black/African American participants had higher odds than White participants (aOR = 1.802, 95% CI: 1.703 to 1.907); the association for Asian/Other/Unknown race was not statistically significant (aOR = 0.959, 95% CI: 0.890 to 1.032).

**Table 3:** Adjusted generalized estimating equation (GEE) results for obesity across the Pre-Pandemic, Pandemic Peak, and Peri-Pandemic periods (N = 38,632).

Variable	Adjusted odds ratio	95% CI	p-value
**Age**			
Younger adults (<50 years)	Reference		
Older adults (≥50 years)	0.889	(0.847, 0.933)	<0.001
**Sex**			
Male	Reference		
Female	1.222	(1.166, 1.281)	<0.001
**Race**			
White	Reference		
Black/African American	1.802	(1.703, 1.907)	<0.001
Asian/Other/Unknown	0.959	(0.890, 1.032)	0.262
**Ethnicity**			
Non-Hispanic/Latino	Reference		
Hispanic/Latino	1.195	(1.103, 1.294)	<0.001
Other/Unknown	0.982	(0.855, 1.129)	0.801
**Annual household income**			
<$25,000	Reference		
$25,000-$50,000	1.042	(0.972, 1.116)	0.249
$50,000-$100,000	0.776	(0.728, 0.828)	<0.001
>$100,000	0.454	(0.426, 0.485)	<0.001
Unknown	0.713	(0.671, 0.758)	<0.001
**Employment status**			
Employed	Reference		
Unemployed	1.009	(0.961, 1.060)	0.715
Time	1.053	(1.039, 1.067)	<0.001
Time squared	0.996	(0.994, 0.997)	<0.001
**Time interactions**			
Older adults × Time	0.965	(0.958, 0.971)	<0.001
Female × Time	1.007	(1.000, 1.014)	0.042
Unemployed × Time	0.985	(0.979, 0.992)	<0.001

Time × subgroup interaction terms were tested for all sociodemographic factors; only statistically significant interactions are presented.

Hispanic/Latino participants had higher odds of obesity than Non-Hispanic/Latino participants (aOR = 1.195, 95% CI: 1.103 to 1.294). Higher household income was associated with lower odds of obesity. Compared with participants earning < $25,000 annually, those earning $50,000–$100,000 had lower odds of obesity (aOR = 0.776, 95% CI: 0.728 to 0.828), with a larger difference among those earning > $100,000 (aOR = 0.454, 95% CI: 0.426 to 0.485). The $25,000–$50,000 group did not differ significantly from the < $25,000 group (aOR = 1.042, 95% CI: 0.972 to 1.116). Employment status was not significantly associated with obesity (aOR = 1.009, 95% CI: 0.961 to 1.060).

#### Time effects.

The linear time term was associated with higher obesity odds (aOR = 1.053, 95% CI: 1.039 to 1.067), whereas the quadratic term was below 1 (aOR = 0.996, 95% CI: 0.994 to 0.997), indicating a nonlinear temporal pattern (Table 3).

#### Interaction effects.

Older adults had smaller changes in obesity odds over time than younger adults (aOR = 0.965, 95% CI: 0.958 to 0.971), and unemployed participants had smaller changes than employed participants (aOR = 0.985, 95% CI: 0.979 to 0.992). The Female × Time interaction was small but statistically significant (aOR = 1.007, 95% CI: 1.000 to 1.014).

### Sensitivity analysis

Findings from the expanded cohort were broadly consistent with the primary analysis, although several individual associations and interaction terms changed statistical significance (S1 Table).

## Discussion

This longitudinal analysis identified modest temporal changes in BMI and obesity across the Pre-Pandemic, Pandemic Peak and Peri-Pandemic periods. Mean BMI increased by 0.09 units at the Pandemic Peak and subsequently declined by 0.18 units, while obesity prevalence increased by 0.3 percentage points and then declined by 0.6 percentage points. Although statistically significant, these absolute changes were modest.

The descriptive age patterns differed between younger and older adults. Obesity prevalence among adults younger than 50 increased during the Pandemic Peak and remained slightly elevated in the Peri-Pandemic phase, whereas older adults showed declines across the three periods. These differences may reflect varied behavioral and healthcare contexts across age groups and are consistent with studies reporting heterogeneous changes in physical activity and dietary habits during the pandemic [31,32]. The younger group includes many reproductive-age adults, for whom weight trajectories may also have intergenerational relevance because parental and preconception BMI have been associated with later offspring risk [33].

Sex differences were also notable in our descriptive trends. Men showed a steady decline in obesity across all three periods, whereas women experienced an increase during the Pandemic Peak period followed by a modest decrease in the Peri-Pandemic period, while still maintaining a higher overall obesity burden compared to men. These patterns align with prior evidence reporting greater weight increases and dietary changes among women and younger adults during the pandemic [34,35].

Adjusted differences in BMI and obesity were evident across sociodemographic groups. Female, Black/African American, and Hispanic/Latino participants, and participants with lower income generally had higher BMI or greater odds of obesity than their reference groups. Changes over time were not uniform: age, sex, and employment interactions were observed for both outcomes, while additional BMI interactions were observed for Black/African American race and ethnicity.

The increase in BMI during the Pandemic Peak is consistent with prior literature describing reduced physical activity, greater sedentary behavior, dietary changes, and psychological stress during periods of lockdown and disruption [16,36,37]. These mechanisms are plausible explanations at the population level, but they were not directly measured in this study and cannot be inferred causally from the temporal associations. The subsequent declines in mean BMI and obesity prevalence may be consistent with partial normalization of routines as restrictions eased, as suggested by survey-based studies [38], although other changes occurring during the same period may also have contributed.

Our findings reaffirm disparities in BMI and obesity associated with race, ethnicity, sex, and income. These patterns predated the pandemic and occurred against longstanding structural inequities in access to healthcare, nutritious food, safe environments for physical activity, and economic stability [39–43]. The persistence of these adjusted disparities across the study periods emphasizes the continuing importance of addressing the social and structural conditions that shape obesity risk, particularly among populations already experiencing disproportionate burden.

The higher adjusted BMI and obesity burden observed in populations experiencing structural disadvantage supports maintaining access to affordable nutritious food, community nutrition programs, safe opportunities for physical activity, and mental health resources during future public-health disruptions. These findings highlight the importance of incorporating equity-focused strategies into preparedness efforts to reduce the risk that future public-health disruptions further disadvantage populations already experiencing a disproportionate burden of obesity.

Findings of this study should be interpreted with caution due to several limitations. First, although the All of Us cohort is large and diverse, it is not representative of the U.S. population. Requiring BMI data in all three periods may preferentially select participants with more continuous healthcare engagement, chronic conditions, obesity, or better access to care, further limiting generalizability. To examine whether this requirement materially influenced the findings, we repeated the analyses in an expanded cohort requiring BMI data in at least two of the three periods. The overall pattern of findings was broadly similar, although participants with more consistent healthcare engagement may still be over-represented. Second, race and ethnicity data also lack subgroup detail, and the Other/Unknown ethnicity group was small; residual misclassification and limited precision are therefore possible. Third, BMI was derived from EHR-recorded height and weight and may be influenced by encounter frequency, data-source variation, and occasional patient-entered measurements. BMI also does not measure body composition. Fourth, income and employment were drawn from baseline survey responses because repeated updates were not consistently available. Fifth, physical activity, stress, COVID-19 infection, anti-obesity medication use, and bariatric surgery were not reliably available for most participants. Sixth, area-level deprivation could not be derived because only three-digit ZIP codes are available, limiting our ability to contextualize neighborhood-level risk. Finally, the broad national period definitions do not capture state-level variation in restrictions, reopening, and recovery.

In future studies, we may further incorporate physical activity (Fitbit) data, stress/coping surveys, COVID-19 infection information, anti-obesity medication use, and bariatric surgery to better evaluate behavioral and clinical contributors to BMI and obesity patterns. Additionally, examining class-specific obesity trajectories (Class I, II, and III) represents an important direction for future research.

## Conclusions

BMI and obesity increased during the Pandemic Peak and subsequently declined during the Peri-Pandemic period. Persistent sociodemographic differences were evident, with higher BMI and/or greater odds of obesity among female, Black/African American and Hispanic/Latino participants, and participants with lower household income. These findings highlight the importance of equity-focused obesity prevention and maintaining access to supportive resources for populations experiencing greater obesity burden during future public-health disruptions.

## Supporting information

S1 Table Comparison of directly comparable adjusted associations in the primary cohort and expanded sensitivity cohort. (DOCX)

## Abbreviations

AoU: All of Us Research Program
BMI: Body Mass Index
COVID-19: Coronavirus Disease 2019
EHR: Electronic Health Record
GEE: Generalized Estimating Equations
IRB: Institutional Review Board
SD: Standard Deviation
aOR: Adjusted Odds Ratio
β (Beta coefficient): Regression Coefficient
WHO: World Health Organization
