# Supplementary material for: Longitudinal changes in BMI and obesity across COVID-19 pandemic periods among underrepresented populations: An analysis of the All of Us dataset
Source: PLoS One. 2026 Oct 27;21(10):e0360745. doi: 10.1371/journal.pone.0360745 (PMC13712593; doi:10.1371/journal.pone.0360745)
Supplement: Supplementary file 1 [file pone.0360745.s001.docx]

**S1 Table.** Comparison of adjusted associations in the primary cohort and expanded sensitivity cohort

The primary cohort included participants with BMI data in all three study periods; the expanded sensitivity cohort included participants with BMI data in at least two of the three periods. Only estimates with directly comparable category definitions are presented.

**Panel A. BMI**

| **Variable** | **Primary cohort (N = 38,632)** | | | **Expanded sensitivity cohort (N = 83,698)** | | |
| --- | --- | --- | --- | --- | --- | --- |
|  | **β** | **95% CI** | **p-value** | **β** | **95% CI** | **p-value** |
| **Age** | | | | | | |
| Younger adults (<50 years) | Reference |  |  | Reference |  |  |
| Older adults (≥50 years) | -0.540 | (-0.684, -0.396) | <0.001 | -0.444 | (-0.543, -0.345) | <0.001 |
| **Sex** | | | | | | |
| Male | Reference |  |  | Reference |  |  |
| Female | 0.523 | (0.383, 0.662) | <0.001 | 0.699 | (0.603, 0.795) | <0.001 |
| **Race** | | | | | | |
| White | Reference |  |  | Reference |  |  |
| Black/African American | 2.461 | (2.265, 2.656) | <0.001 | 2.360 | (2.226, 2.495) | <0.001 |
| Asian/Other/Unknown | -0.220 | (-0.471, 0.030) | 0.085 | -0.470 | (-0.634, -0.306) | <0.001 |
| **Annual household income** | | | | | | |
| <$25,000 | Reference |  |  | Reference |  |  |
| $25,000-$50,000 | -0.035 | (-0.277, 0.206) | 0.775 | 0.129 | (-0.038, 0.296) | 0.129 |
| $50,000-$100,000 | -1.064 | (-1.287, -0.841) | <0.001 | -0.872 | (-1.027, -0.717) | <0.001 |
| >$100,000 | -2.907 | (-3.125, -2.688) | <0.001 | -2.565 | (-2.717, -2.413) | <0.001 |
| Unknown | -1.389 | (-1.602, -1.176) | <0.001 | -1.263 | (-1.408, -1.119) | <0.001 |
| **Employment status** | | | | | | |
| Employed | Reference |  |  | Reference |  |  |
| Unemployed | 0.037 | (-0.112, 0.185) | 0.628 | 0.213 | (0.110, 0.317) | <0.001 |
| **Time terms** | | | | | | |
| Time | 0.218 | (0.192, 0.243) | <0.001 | 0.216 | (0.196, 0.236) | <0.001 |
| Time squared | -0.018 | (-0.021, -0.015) | <0.001 | -0.017 | (-0.019, -0.014) | <0.001 |
| **Time interactions** | | | | | | |
| Older adults × Time | -0.139 | (-0.148, -0.130) | <0.001 | -0.143 | (-0.151, -0.136) | <0.001 |
| Female × Time | 0.020 | (0.011, 0.029) | <0.001 | 0.024 | (0.017, 0.031) | <0.001 |
| Unemployed × Time | -0.057 | (-0.067, -0.048) | <0.001 | -0.054 | (-0.062, -0.047) | <0.001 |
| Black/African American × Time | -0.043 | (-0.056, -0.031) | <0.001 | -0.035 | (-0.045, -0.025) | <0.001 |
| Asian/Other/Unknown race × Time | 0.003 | (-0.013, 0.020) | 0.691 | 0.000 | (-0.012, 0.012) | 0.990 |
| $25,000-$50,000 × Time | 0.001 | (-0.014, 0.017) | 0.862 | -0.007 | (-0.020, 0.005) | 0.240 |
| $50,000-$100,000 × Time | 0.005 | (-0.009, 0.020) | 0.471 | 0.000 | (-0.012, 0.011) | 0.983 |
| >$100,000 × Time | 0.003 | (-0.011, 0.018) | 0.639 | -0.002 | (-0.014, 0.009) | 0.691 |
| Unknown income × Time | -0.012 | (-0.025, 0.002) | 0.102 | -0.012 | (-0.023, -0.001) | 0.028 |

**Panel B. Obesity**

| **Variable** | **Primary cohort (N = 38,632)** | | | **Expanded sensitivity cohort (N = 83,698)** | | |
| --- | --- | --- | --- | --- | --- | --- |
|  | **aOR** | **95% CI** | **p-value** | **aOR** | **95% CI** | **p-value** |
| **Age** | | | | | | |
| Younger adults (<50 years) | Reference |  |  | Reference |  |  |
| Older adults (≥50 years) | 0.889 | (0.847, 0.933) | <0.001 | 0.921 | (0.891, 0.953) | <0.001 |
| **Sex** | | | | | | |
| Male | Reference |  |  | Reference |  |  |
| Female | 1.222 | (1.166, 1.281) | <0.001 | 1.285 | (1.243, 1.328) | <0.001 |
| **Race** | | | | | | |
| White | Reference |  |  | Reference |  |  |
| Black/African American | 1.802 | (1.703, 1.907) | <0.001 | 1.788 | (1.721, 1.858) | <0.001 |
| Asian/Other/Unknown | 0.959 | (0.890, 1.032) | 0.262 | 0.892 | (0.851, 0.936) | <0.001 |
| **Annual household income** | | | | | | |
| <$25,000 | Reference |  |  | Reference |  |  |
| $25,000-$50,000 | 1.042 | (0.972, 1.116) | 0.249 | 1.058 | (1.009, 1.109) | 0.002 |
| $50,000-$100,000 | 0.776 | (0.728, 0.828) | <0.001 | 0.819 | (0.784, 0.856) | <0.001 |
| >$100,000 | 0.454 | (0.426, 0.485) | <0.001 | 0.497 | (0.475, 0.519) | <0.001 |
| Unknown | 0.713 | (0.671, 0.758) | <0.001 | 0.736 | (0.707, 0.767) | <0.001 |
| **Employment status** | | | | | | |
| Employed | Reference |  |  | Reference |  |  |
| Unemployed | 1.009 | (0.961, 1.060) | 0.715 | 1.040 | (1.005, 1.077) | 0.025 |
| **Time terms** | | | | | | |
| Time | 1.053 | (1.039, 1.067) | <0.001 | 1.054 | (1.043, 1.065) | <0.001 |
| Time squared | 0.996 | (0.994, 0.997) | <0.001 | 0.996 | (0.995, 0.997) | <0.001 |
| **Time interactions** | | | | | | |
| Older adults × Time | 0.965 | (0.958, 0.971) | <0.001 | 0.962 | (0.957, 0.967) | <0.001 |
| Female × Time | 1.007 | (1.000, 1.014) | 0.042 | 1.006 | (1.001, 1.012) | 0.015 |
| Unemployed × Time | 0.985 | (0.979, 0.992) | <0.001 | 0.988 | (0.983, 0.993) | <0.001 |

*Note.* Adjusted β coefficients for BMI from linear mixed-effects models and adjusted odds ratios (aORs) for obesity from generalized estimating equation models are presented.

Reference categories are indicated by 'Reference';

Abbreviations: aOR, adjusted odds ratio; BMI, body mass index; CI, confidence interval.
